# Canine rabies control and human exposure 1951–2015, Guangzhou, China

**DOI:** 10.2471/BLT.18.217372

**Published:** 2018-11-27

**Authors:** Yuehong Wei, Xiaoning Liu, Dapeng Li, Shouyi Chen, Jianmin Xu, Kuncai Chen, Zhicong Yang

**Affiliations:** a Guangzhou Center for Disease Control and Prevention, No. 1 Qi De road, Baiyun District, Guangzhou, China.; b School of Public Health, Guangdong Pharmaceutical University, Guangzhou, China.

## Abstract

**Objective:**

To describe changes in rabies surveillance and management in Guangzhou, China between 1951 and 2015, and to analyse human rabies cases over that period.

**Methods:**

Rabies control policies and strategies implemented by the Guangzhou government were reviewed for three periods: 1951 to 1978, 1979 to 2000 and 2001 to 2015. Data on human rabies deaths and exposure were obtained from Guangzhou and national health and disease records. The demographic characteristics of human cases are reported using descriptive statistics.

**Findings:**

Between 1951 and 2015, the number of organizations cooperating on rabies control increased: there were two between 1951 and 1978, six between 1979 and 2000, and nine between 2001 and 2015. The number of human rabies cases reported in these periods was 331, 422 and 60, respectively. Organizations involved included city and district centres for disease control and prevention, rabies outpatient clinics, medical institutions and police. Overall, 88% (713/813) of cases occurred in rural districts, though, between 1951 and 2015, the distribution shifted from being predominantly rural to being both urban and rural. The number of people exposed to rabies increased annually. The biggest increases were among those injured by a pet dog or other animal: 3.26 and 4.75 times, respectively, between 2005 and 2015.

**Conclusion:**

Increased cooperation on rabies control between civil organizations in Guangzhou over decades was associated with a marked decrease in the number of human rabies cases. The Guangzhou experience could thereby provide guidance for other cities experiencing similar rabies epidemics.

## Résumé

**Objectif:**

Décrire l'évolution de la surveillance et de la gestion de la rage à Guangzhou, en Chine, entre 1951 et 2015, et analyser les cas de rages humaines survenus au cours de cette période.

**Méthodes:**

Les politiques et stratégies de contrôle de la rage mises en œuvre par le gouvernement de Guangzhou ont été examinées au regard de trois périodes: de 1951 à 1978, de 1979 à 2000 et de 2001 à 2015. Les données relatives aux décès et à l’exposition des personnes à la rage proviennent des dossiers de santé et sur les maladies de Guangzhou et du pays. Les caractéristiques démographiques des cas humains sont présentées à l'aide de statistiques descriptives.

**Résultats:**

Entre 1951 et 2015, le nombre d'organisations agissant en coopération pour contrôler la rage a augmenté: elles étaient au nombre de deux entre 1951 et 1978, six entre 1979 et 2000, et neuf entre 2001 et 2015. Le nombre de cas de rage signalés au cours de ces périodes s'élevait respectivement à 331, 422 et 60. Les organisations en question incluaient des centres de prévention et de contrôle de la maladie au niveau des villes et des districts, des dispensaires pour patients ambulatoires atteints de la rage, des institutions médicales et la police. En tout, 88% (713/813) des cas sont survenus dans des districts ruraux. Néanmoins, entre 1951 et 2015, la répartition a cessé d'être principalement rurale pour devenir aussi bien urbaine que rurale. Le nombre de personnes exposées à la rage s'est accru d'année en année. Les plus fortes hausses ont concerné les personnes blessées par un chien de compagnie ou un autre animal: 3,26 et 4,75 fois, respectivement, entre 2005 et 2015.

**Conclusion:**

Le renforcement de la coopération en matière de contrôle de la rage entre diverses organisations civiles à Guangzhou depuis plusieurs décennies a été associé à une nette diminution du nombre de cas de rage. L'expérience de Guangzhou pourrait donc donner des orientations à d'autres villes touchées par des épidémies de rage similaires.****

## Resumen

**Objetivo:**

Describir los cambios en la vigilancia y la gestión de la rabia en Guangzhou, China, entre 1951 y 2015 y analizar los casos de rabia en humanos durante ese periodo.

**Métodos:**

Las políticas y estrategias de control de la rabia aplicadas por el gobierno de Guangzhou se revisaron durante tres periodos: De 1951 a 1978, de 1979 a 2000 y de 2001 a 2015. Se recopilaron los datos sobre las muertes y la exposición a la rabia en humanos en Guangzhou y sobre los registros nacionales de salud y enfermedad. Las características demográficas de los casos en humanos se registran mediante estadísticas descriptivas.

**Resultados:**

Entre 1951 y 2015, el número de organizaciones que cooperaban en el control de la rabia aumentó: hubo dos entre 1951 y 1978, seis entre 1979 y 2000, y nueve entre 2001 y 2015. El número de casos de rabia notificados en estos periodos fue de 331, 422 y 60, respectivamente. Entre las organizaciones participantes figuraban los centros urbanos y de distrito para el control y la prevención de enfermedades, las clínicas ambulatorias para el control de la rabia, las instituciones médicas y la policía. En total, el 88 % (713/813) de los casos tuvieron lugar en distritos rurales, aunque, entre 1951 y 2015, la distribución pasó de ser predominantemente rural a ser tanto urbana como rural. El número de personas expuestas a la rabia aumentó anualmente. Los mayores aumentos se produjeron entre los heridos por un perro doméstico u otro animal: 3,26 y 4,75 veces, respectivamente, entre 2005 y 2015.

**Conclusión:**

El aumento de la cooperación en el control de la rabia entre las organizaciones civiles de Guangzhou durante décadas se asoció con una marcada reducción del número de casos de rabia. La experiencia de Guangzhou podría servir de guía a otras ciudades que sufren epidemias de rabia similares.

## ملخص

**الغرض:**

لوصف التغييرات في مراقبة داء الكلب وإدارته في قوانغتشو، بالصين بين عامي 1951 و2015 وتحليل حالات السعار البشري خلال تلك الفترة.

**الطريقة:**

تمت مراجعة سياسات واستراتيجيات السيطرة على داء الكلب التي نفذتها حكومة قوانغتشو لمدة ثلاث فترات: 1951 إلى 1978، و1979 إلى 2000، و2001 حتى 2015. تم الحصول على بيانات عن وفيات حالات داء السعار البشري والتعرض له، من قوانغتشو وسجلات المرض والصحة الوطنية. يتم الإبلاغ عن الخصائص السكانية للحالات البشرية باستخدام الإحصاء الوصفي.

**النتائج:**

بين عامي 1951 و2015، زاد عدد المنظمات التي تتعاون في مكافحة داء الكلب: كان هناك اثنتان بين عامي 1951 و1978، وستة بين عامي 1979 و2000، وتسعة بين عامي 2001 و2015. عدد حالات داء الكلب التي تم الإبلاغ عنها في هذه الفترات هو 331 و422 و60 على الترتيب. وشملت المنظمات المعنية مراكز المدن والمقاطعات لمكافحة الأمراض والوقاية منها، والعيادات الخارجية لداء الكلب، والمؤسسات الطبية والشرطة. بشكل عام، وقعت 88٪ (713/813) من الحالات في المناطق الريفية، وعلى الرغم من ذلك، انتقل التوزيع بين عامي 1951 و2015 من كونه ريفي في الغالب إلى كونه حضريًا وريفيًا. وكان عدد الأشخاص الذين يتعرضون لداء الكلب يزداد سنوياً. وكانت أكبر الزيادات بين هؤلاء المصابين بواسطة كلب أليف أو حيوان غيره: 3.26 و4.75 مرة على الترتيب، بين عامي 2005 و2015.

**الاستنتاج:**

ارتبطت زيادة التعاون في مكافحة داء الكلب بين المنظمات المدنية في قوانغتشو على مدى عقود مع انخفاض ملحوظ في عدد حالات داء الكلب. وبالتالي يمكن أن توفر تجربة قوانغتشو توجيهات للمدن الأخرى التي تعاني من أوبئة مماثلة لداء الكلب. ****

## 摘要

**目的:**

旨在描述 1951 年至 2015 年，中国广州在狂犬病监测及管理方面做出的改变，并对该时期的人类狂犬病病例进行分析。

**方法:**

广州政府实施的狂犬病控制政策和策略经过了三个时期：1951 年至 1978 年，1979 年至 2000 年以及 2001 年至 2015 年。人类狂犬病死亡和感染数据来源于广州和国家健康和疾病中心的相关记录。使用描述统计学报告人类狂犬病病例的人口特征。

**结果:**

1951 年至 2015 年，参与狂犬病控制的合作机构数量得以增加，分别是：1951 年至 1978 年 2 家；1979 年至 2000 年 6 家，2001 年至 2015 年 9 家，报告的狂犬病病例数量分别为 331 起、422 起和 60 起。涉及组织包括城市和地区的疾病控制和预防中心、狂犬病门诊、医疗机构和警方。总体上，88%（813 中有 713 起）的病例发生在农村地区，但在 1951 年至 2015 年，狂犬病分布从主要在农村转变为城市和农村均有。感染狂犬病的人数每年都在增加。增幅最大的群体是遭到宠物狗或其他动物伤害的人：2005 年至 2015 年，增幅分别为 3.26 倍和 4.75 倍。

**结论:**

几十年来，与广州民间组织加强狂犬病控制的合作与狂犬病病例数量的显著下降有关。因此，广州的经验可以为其他正经历类似流行性狂犬病困扰的城市提供指导。****

## Резюме

**Цель:**

Описать изменения в эпиднадзоре и лечении бешенства в Гуанчжоу, Китай, в период с 1951 по 2015 год и проанализировать случаи заболевания бешенством в этот период.

**Методы:**

Были исследованы реализованные правительством Гуанчжоу политика и стратегии контроля бешенства в течение трех периодов: с 1951 по 1978 год, с 1979 по 2000 год и с 2001 по 2015 год. Данные о смертности и случаях заражения бешенством людей были получены из статистических данных о заболеваемости Гуанчжоу и национального архива. Демографические характеристики случаев заболевания человека указаны на основе описательной статистики.

**Результаты:**

В период с 1951 по 2015 год увеличилось число организаций, задействованных для контроля над бешенством: между 1951 и 1978 годами было две такие организации, с 1979 по 2000 год — шесть, с 2001 по 2015 год — девять. Количество случаев заболевания бешенством, зарегистрированных в эти периоды, составляло соответственно 331, 422 и 60. Задействованные организации включали городские и районные центры эпиднадзора и профилактики заболеваний, амбулаторные поликлиники, медицинские учреждения и полицию. В целом 88% случаев (713/813) были зарегистрированы в сельских районах, хотя между 1951 и 2015 годами их распространение изменилось с преимущественно сельских районов на городские и сельские. Количество инфицированных бешенством людей ежегодно увеличивалось. Наибольший рост был среди людей, укушенных домашней собакой или другим животным: в 3,26 и 4,75 раза соответственно в период с 2005 по 2015 год.

**Вывод:**

Расширение сотрудничества между гражданскими организациями по контролю над бешенством в Гуанчжоу на протяжении десятилетий привело к заметному сокращению количества случаев заболевания бешенством. Таким образом, опыт Гуанчжоу может служить руководством для других городов со сходной эпидемической ситуацией по бешенству.****

## Introduction

Rabies is a fatal, zoonotic, disease caused by an RNA virus of the genus *Lyssavirus*.^1^ Almost all mammals are susceptible to infection,^2^^,^^3^ which can occur via bites or scratches from infected animals or through contamination of fresh wounds or mucous membranes by infectious material.^4^ In humans, the fatality rate is almost 100%.^5^^–^^8^ Worldwide, rabies is a commonly neglected zoonotic disease, especially in developing countries. Mainland China has the second highest rabies incidence in the world, after India^6^^–^^8^ and the impact on public health is substantial.^9^^,^^10^ In China, rabies is the third leading cause of death from notifiable diseases, behind acquired immune deficiency syndrome and tuberculosis.^11^

The threat of rabies is considerable in many countries around the world. Several of these countries have responded proactively to a proposal by the World Health Organization (WHO) to eliminate rabies transmission from dogs to humans before 2030, by exploring different ways of eradicating endemic rabies.^9^ One way is to apply a cooperative effort across multiple disciplines at local, national and global levels that aims to achieve the best results for health by taking into account people, animals and the environment.^12^^–^^14^ The underlying rationale is that disease, particularly zoonotic disease, is influenced by human, animal and environmental factors and can only be tackled through better interdisciplinary and institutional communication, cooperation and collaboration. Applying a cooperative approach reduces health risks to both humans and animals. In practice, this approach involves the combined efforts of public health professionals, doctors and veterinary physicians, as well as staff in related disciplines and institutions. The approach has been applied in Africa, India and Latin America and as a result, the threat of rabies in these areas has decreased.^15^ In addition, countries that have successfully eliminated rabies, such as Spain,^16^ have adopted a cooperative approach to managing imported cases of rabies.

During the last century, rabies has been endemic in animals in the city of Guangzhou, China and local government has implemented several strategies for controlling the disease and preventing human infection. As techniques for control and prevention have improved and an increasing number of organizations have become involved, human rabies cases have decreased. The aim of this paper was to review the rabies control and prevention strategies implemented in Guangzhou in recent decades to provide guidance for other cities confronting a similar challenge.

## Methods

**Fig. 1:**
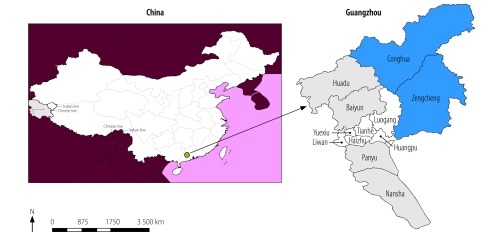
Administrative divisions, Guangzhou, China, 2018

Note: The city of Guangzhou comprises six urban districts (white), four rural districts (grey) and two satellite towns (blue).

Guangzhou, the capital city of Guangdong, is located in the south of China (Fig. 1). The city covers an area of 7473.4 km^2^, which includes two satellite towns (Zengcheng and Conghua), six urban administrative districts (Huangpu, Tianhe, Yuexiu, Liwan, Haizhu and Luogan) and four rural administrative districts (Panyu, Huadu, Baiyun and Nansha). The population was around 1.38 million in 1951 and 13.5 million in 2015. In 1990, the annual per capita disposable income in rural and urban areas was 1539 yuan (221 United States dollars, US$) and 2749 yuan (US$ 395), respectively and in 2015, it was 17 663 yuan (US$ 2543) and 42 955 yuan (US$ 6185), respectively.

Source of shapefile: Guangzhou Bureau of Statistics, Guangzhou, China.

**Fig. 2:**
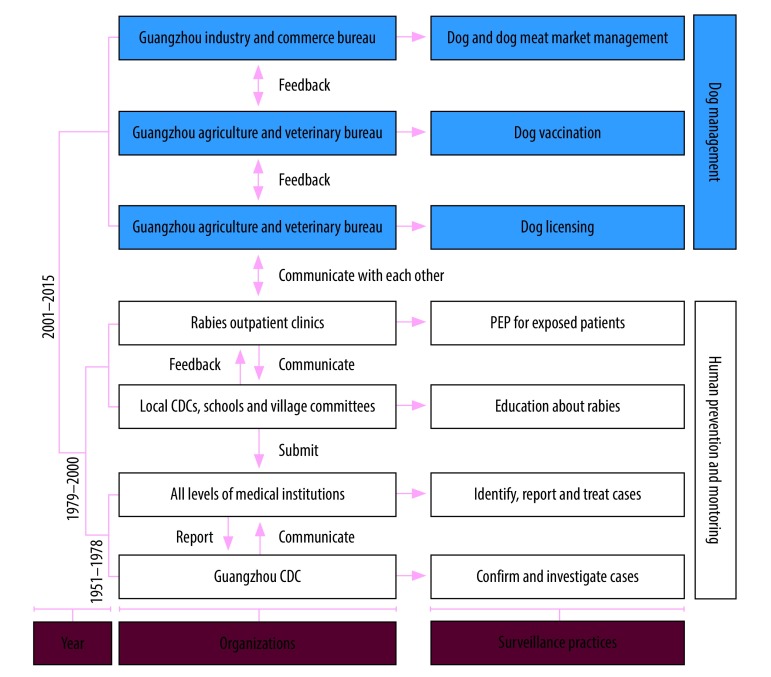
Development of rabies control system, by period, Guangzhou, China, 1951–2015

CDC: centre for disease control and prevention; PEP: postexposure prophylaxis.

The current system of rabies surveillance and management in Guangzhou has been developed and improved over the decades and comprises two parts: human prevention and monitoring; and dog management (Fig. 2). The Guangzhou Center for Disease Control and Prevention is primarily in charge of confirming the diagnosis of rabies in humans and of carrying out epidemiological investigations of rabies cases, leaving other institutions responsible for rabies control work. Medical facilities at every level and rabies outpatient clinics are responsible for identifying cases and offering postexposure prophylaxis (i.e. four doses of rabies vaccine and rabies immunoglobulin) to people who have been bitten or scratched by dogs, cats or other animals. These institutions also record the details of exposed people when they seek medical help. Local centres for disease control and prevention, schools and village committees are responsible for educating people susceptible to rabies infection, the local police are responsible for licensing dogs, the Guangzhou agriculture and veterinary bureau is responsible for vaccinating dogs and the Guangzhou industry and commerce bureau is responsible for regulating the dog and dog meat trade. There is extensive, regular cooperation and communication between these organizations.

### Data analysis

All human rabies deaths reported in this study are confirmed cases. Data on rabies cases that occurred between 1951 and 2004 were obtained from the Guangzhou yearbook of health statistics (Guangzhou Center for Disease Control and Prevention, unpublished data, 2018) and data on cases between 2005 and 2015 were obtained from the National Disease Reporting Information System (Chinese Center for Disease Control and Prevention, unpublished data, 2018). Data on patients with confirmed rabies who received postexposure prophylaxis between 2005 and 2015 were obtained from surveillance sites located at rabies outpatient clinics. Human rabies was diagnosed according to WHO’s expert consultation on rabies.^17^ The criteria for a probable case of rabies were a history of exposure (i.e. contact with a suspected rabid animal) and the presence of a clinical syndrome characterized by excitability, hydrophobia, fear of drafts and pharyngeal muscle spasms. 

In addition to meeting these criteria, confirmed rabies cases also had to have at least one of the following laboratory findings: (i) the presence of rabies virus antigens; (ii) isolation of virus in cell culture or a laboratory animal; (iii) the presence of viral-specific antibodies in the cerebrospinal fluid or serum of an unvaccinated person; or (iv) the detection of viral nucleic acids by molecular methods in a postmortem or *intra vitam* sample of, for example, brain tissue, skin, saliva or concentrated urine. In some cases, there was no clinical suspicion of encephalitis or no history of animal exposure, but the case was confirmed by laboratory diagnostic tests. Initial data were entered into a database using EpiData Entry v. 3.1 (EpiData, Odense, Denmark), then analysed and depicted using R v. 3.31 (The R Foundation, Vienna, Austria). The demographic characteristics of human cases are presented using descriptive statistics.

## Results

### Development of rabies control

Between 1951 and 1978, human rabies was a nationally notifiable infectious disease in China and doctors at every level of medical institutions in Guangzhou had to report suspected or confirmed cases to the Guangzhou Center for Disease Control and Prevention within 24 to 48 hours. Subsequently, patients were given a second and confirmatory diagnosis by the Center for Disease Control and Prevention. During this time period, only two types of organization were involved in the management of rabies: medical institutions and the Guangzhou Center for Disease Control and Prevention (Fig. 2). Rabies control involved killing the infected dog, epidemiological investigation, postexposure prophylaxis and rabies education in the local area.

**Fig. 3:**
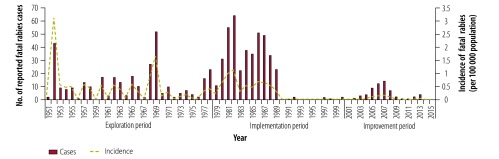
Human rabies cases, Guangzhou, China, 1951–2015

Between 1979 and 2000, after a human rabies case had been reported, everyone closely connected to the patient would be immunized, all dogs within 2.5 km of the place where the case occurred would be killed and it would be forbidden to be keep or sell dogs in that area for 1 year. At the same time, an extensive educational programme about rabies would be conducted in schools, communities and villages. Moreover, in the late 1980s, the Chinese government established rabies outpatient clinics, where exposed people could get postexposure prophylaxis much more easily (Fig. 2). These clinics dealt only with rabies and were located in district centres for disease control and prevention, hospitals and community health centres. During this time period, six types of organization were involved in the management and surveillance of rabies: district centres for disease control and prevention, schools, village committees and rabies outpatient clinics, as well as medical institutions and the Guangzhou Center for Disease Control and Prevention. In addition, since the Cultural Revolution between 1966 and 1976, many health workers, so called barefoot doctors, have been introduced into the countryside and public health services have been provided with more doctors, medicines and medical equipment. Barefoot doctors raised awareness of rabies prevention and treatment among the population and helped increase treatment and reporting rates after rabies exposure. As a result of these improvements, rabies diagnostic and monitoring capacity improved markedly. Moreover, since economic reforms started in 1978, people's living standards have risen continuously and awareness of disease prevention has increased. Together with rabies prevention measures, these changes have had a considerable effect: only one human rabies case was reported in 1991 and none was reported between 1993 and 1996 (Fig. 3).

Between 2001 and 2015, almost all of the 125 rabies outpatient clinics in Guangzhou started to be used as monitoring points for a rabies monitoring system. The local police started to restrict dog density through dog licensing. The Guangzhou agriculture and veterinary bureau started to immunize dogs by injection at regular intervals and the Guangzhou industry and commerce bureau started to limit the trade in dogs and dog meat products, especially in rabies endemic areas (Fig. 2). In addition, in response to a rabies epidemic in 2005, a dog management regulation and a code of practice for the prevention and treatment of rabies exposure were introduced in Guangzhou.^18^^,^^19^ In 2016, the Chinese health ministry issued a rabies control and prevention guide,^20^ according to which the Guangzhou Center for Disease Control and Prevention had to establish a rabies surveillance and management system in cooperation with eight other organizations. These included: (i) the Guangzhou industry and commerce bureau; (ii) the Guangzhou agriculture and veterinary bureau; (iii) local police; (iv) rabies outpatient clinics; (v) local centres for disease control and prevention; (vi) schools; (vii) village committees; and (viii) medical institutions.

### Human rabies cases

Between 1951 and 2015, 813 human rabies cases were recorded in Guangzhou (Fig. 3). The first peak occurred in 1952, with 43 cases. Thereafter, the number of cases was relatively stable for over a decade. Interestingly, there were minor peaks roughly every 3 years between 1956 and 1967, which increased in magnitude. The second outbreak occurred in 1968 and 1969, with 27 and 52 cases in each year, respectively. The largest and longest outbreak lasted for 13 years and began in 1977, with a peak of 64 cases in 1982. During this period, 452 cases were reported, which accounted for over 55% (452/813) of cases in the entire study period. Subsequently, the number of cases decreased dramatically from 373 during the 1980s to 8 during the 1990s and few cases have occurred in the following years. However, this flat trend changed in 2003 for a 6-year period, during which there was a peak of 14 cases in 2007.

**Fig. 4:**
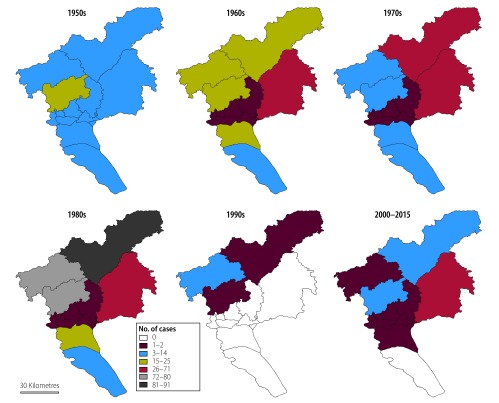
Geographical distribution of human rabies cases, by decade, Guangzhou, China, 1951–2015

Note: The city of Guangzhou comprises six urban districts, four rural districts and two satellite towns, as shown in Fig. 1.

The geographical distribution of human rabies cases in Guangzhou was analysed over the decades between 1951 and 2015 (Fig. 4). Cases occurring in rural areas accounted for 88% (713/813) of all human cases. In the 1950s, the highest number of cases were in Baiyun and Panyu districts: 20 and 12 cases, respectively. In later decades, the highest numbers of human rabies cases tended to occur in northern rural areas, especially in the 1980s. In the 1960s and 1970s, the largest number of cases were found in Zengcheng district: 43 and 34 cases occurred in these two decades, respectively. In the most severe epidemic in the 1980s, the total number of cases in all northern rural areas was 70, less than in Conghua district alone, where there were 90 cases. After this epidemic, only 8 cases were reported in the whole of Guangzhou throughout the entire 1990s and there was no case in any of the six urban districts or in one of the two satellite towns. However, between 2000 and 2015, particularly during the late 2000s, another rabies outbreak occurred. At that time, the number of cases in Zengcheng district equalled that in all other districts combined. In the six urban districts, a total of 13 cases were reported between 2000 and 2015, almost equal to the total reported between the 1960s and 1980s in these districts.

Source of shapefile: Guangzhou Bureau of Statistics, Guangzhou, China.

**Fig. 5:**
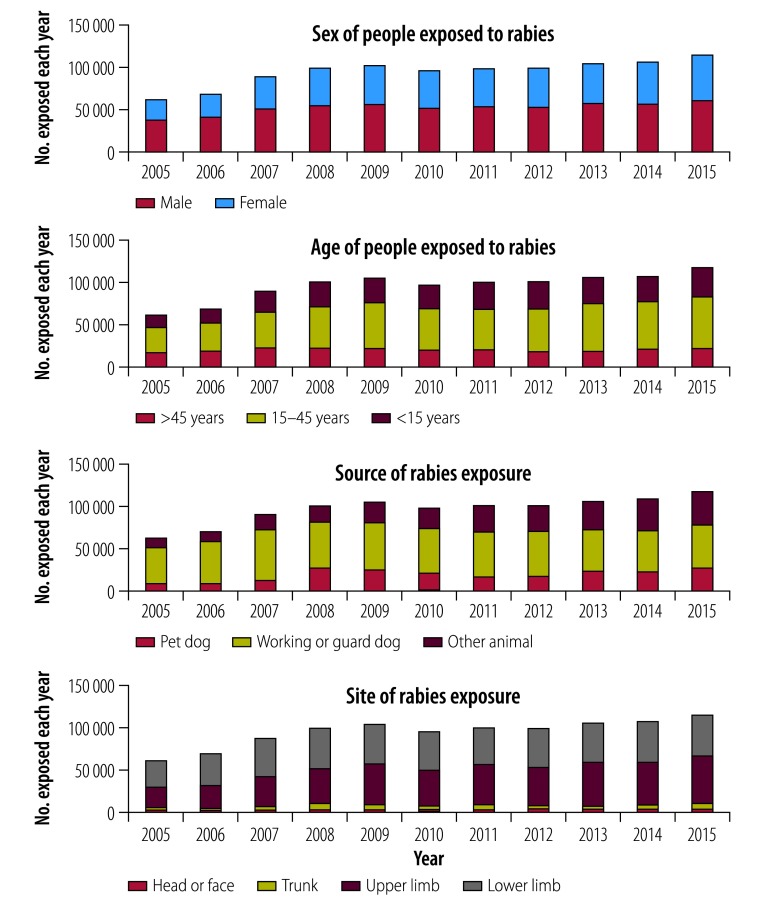
People exposed annually to rabies, by sex, age, animal source and anatomical exposure site, Guangzhou, China, 2005–2015

The number of people exposed to rabies between 2005 and 2015 was 1 050 553, according to data from monitoring sites at rabies outpatient clinics collected by the Guangzhou Center for Disease Control and Prevention (Fig. 5). The number of cases of exposure reported increased annually, such that, by 2015, it was almost double (61 983/116 776) that in 2005. In more than 90% of cases (57 216/61 983), exposure occurred in the upper or lower limbs. In 2005, the ratio of males to females exposed to rabies was 1.39:1; this ratio decreased over a decade to 1.13:1 in 2015. The proportion of exposed individuals who were younger than 15 years decreased from 26% (16 341/61 983) in 2005 to 19% (21 806/116 776) in 2015. However, the proportion older than 45 years increased from 25% (15 758/61 983) in 2005 to 30% (34 728/116 776) in 2015. The biggest change was in the number of exposed people who were injured by a pet dog or other animal: between 2005 and 2015, the number increased 3.26 and 4.75 times, respectively. Rabies exposure from guard dogs remained stable over time.

## Discussion

After decades of battling with rabies, the Guangzhou government has achieved remarkable results and, in 2014 and 2015, there were no cases in the city. The rabies surveillance and management system has been improved and the Guangzhou government has issued strict regulations to reduce canine density, improve postexposure prophylaxis and increase public awareness of rabies prevention. Over the years, the establishment of this system involved the cooperation of an increasing number of organizations, which illustrates the importance of multisectoral collaboration.^21^^,^^22^

Multidisciplinary collaboration is an important step towards the elimination of rabies.^15^ The disease has already been eliminated in many developed countries and even in developing Latin American countries, the incidence of both human and canine rabies has fallen by 90% thanks to help from international organizations and the establishment of governmental committees.^23^ In those countries, rabies control was based on the core strategies of: (i) annual mass vaccination of dogs; (ii) easy access to free postexposure prophylaxis; (iii) disease surveillance; and (iv) education and communication.^21^

In Guangzhou, maintaining the number of rabies cases at zero involves addressing several persistent potential threats. Rabies is mainly caused by dogs in developing countries and by wild animals in developed countries.^24^ However, the main source of infection in rural areas is free-roaming dogs, which may be either guard dogs or pets.^25^ In Guangzhou, efforts to prevent the spread of the rabies virus must consider the changing distribution of rabies cases from predominantly rural areas to both urban and rural areas. According to WHO’s recommendations on the prevention of rabies, the vaccination rate in dogs should be at least 70% but, if this cannot be achieved, postexposure prophylaxis should be used as a last defence against human rabies.^26^^,^^27^ Consequently, despite the usefulness of mass dog vaccination for eliminating rabies,^28^ investment in strategies that reduce the health burden of the disease in humans is also important.^29^^,^^30^

Two other potential threats to rabies control in Guangzhou are a lack of awareness of the disease among the population and limited access to postexposure prophylaxis. In recent years, the number of people exposed to rabies has increased dramatically, particularly the number injured by pets. Some people exposed to infection after being injured by an animal did nothing, because they were not aware that rabies is fatal. One important policy introduced in the 1980s that helped control rabies successfully, aimed at increasing public knowledge about protection against rabies and about the behaviour of rabid dogs. This strategy was particularly beneficial for high-risk sectors of the population, especially children and people living in rural areas.^31^^–^^33^ Research has demonstrated that income plays an important role in gaining access to postexposure prophylaxis. Even in 2010, the annual per capita disposable income of rural residents in Guangzhou was under 18 000 yuan (US$ 2591), whereas the cost of rabies postexposure prophylaxis was approximately 1000 yuan (US$ 144), which made treatment a substantial financial burden for rural residents.^34^ Consequently, new policies should be introduced to make it easier for people exposed to rabies, particularly those living in rural areas, to obtain postexposure prophylaxis.

One limitation of our study was that the number of cases of rabies exposure was probably underestimated, especially in rural areas. Studies have shown that around 50% of exposed people do not seek medical treatment. Consequently, promoting knowledge of rabies among the general population is still very important.^35^

The current rabies control system in Guangzhou took decades to establish and is now very mature and costs around 120 000 yuan (US$ 17 279) per annum to operate. The control effort has had a clear effect on the incidence of rabies. Moreover, the approach demonstrates to other countries and regions with a similar rabies problem that multisectoral collaboration is vital.
